# Resident Willingness to Participate in Digital Contact Tracing in a COVID-19 Hotspot: Findings From a Detroit Panel Study

**DOI:** 10.2196/39002

**Published:** 2023-01-19

**Authors:** Lydia Wileden, Denise Anthony, Celeste Campos-Castillo, Jeffrey Morenoff

**Affiliations:** 1 Mansueto Institute for Urban Innovation University of Chicago Chicago, IL United States; 2 Division of the Social Sciences University of Chicago Chicago, IL United States; 3 Department of Health Management & Policy School of Public Health University of Michigan Ann Arbor, MI United States; 4 School of Information University of Michigan Ann Arbor, MI United States; 5 Department of Sociology University of Michigan Ann Arbor, MI United States; 6 Department of Media & Information Michigan State University East Lansing, MI United States; 7 Gerald R. Ford School of Public Policy University of Michigan Ann Arbor, MI United States; 8 Population Studies Center University of Michigan Ann Arbor, MI United States

**Keywords:** COVID-19, contact tracing, surveillance, informatics, trust, racial disparities

## Abstract

**Background:**

Digital surveillance tools and health informatics show promise in counteracting diseases but have limited uptake. A notable illustration of the limits of such tools is the general failure of digital contact tracing in the United States in response to COVID-19.

**Objective:**

We investigated the associations between individual characteristics and the willingness to use app-based contact tracing in Detroit, a majority-minority city that experienced multiple waves of COVID-19 outbreaks and deaths since the start of the pandemic. The aim of this study was to examine variations among residents in the willingness to download a contact tracing app on their phones to provide public health officials with information about close COVID-19 contact during summer 2020.

**Methods:**

To examine residents’ willingness to participate in digital contact tracing, we analyzed data from 2 waves of the Detroit Metro Area Communities Study, a population-based survey of Detroit, Michigan residents. The data captured 1873 responses from 991 Detroit residents collected in June and July 2020. We estimated a series of multilevel logit models to gain insights into differences in the willingness to participate in digital contact tracing across a variety of individual attributes, including race/ethnicity, degree of trust in the government, and level of education, as well as interactions among these variables.

**Results:**

Our results reflected widespread reluctance to participate in digital contact tracing in response to COVID-19, as less than half (826/1873, 44.1%) of the respondents said they would be willing to participate in app-based contact tracing. Compared to White respondents, Black (odds ratio [OR] 0.45, 95% CI 0.23-0.86) and Latino (OR 0.32, 95% CI 0.11-0.99) respondents were significantly less willing to participate in digital contact tracing. Trust in the government was positively associated with the willingness to participate in digital contact tracing (OR 1.17, 95% CI 1.07-1.27), but this effect was the strongest for White residents (OR 2.14, 95% CI 1.55-2.93). We found similarly divergent patterns of the effects of education by race. While there were no significant differences among noncollege-educated residents, White college-educated residents showed greater willingness to use app-based contact tracing (OR 6.12, 95% CI 1.86-20.15) and Black college-educated residents showed less willingness (OR 0.46, 95% CI 0.26-0.81).

**Conclusions:**

Trust in the government and education contribute to Detroit residents’ wariness of digital contact tracing, reflecting concerns about surveillance that cut across race but likely arise from different sources. These findings point to the importance of a culturally informed understanding of health hesitancy for future efforts hoping to leverage digital contact tracing. Though contact tracing technologies have the potential to advance public health, unequal uptake may exacerbate disparate impacts of health crises.

## Introduction

Throughout the COVID-19 pandemic, public health authorities have engaged in contact tracing to inform those exposed to COVID-19, monitor for signs and symptoms of the virus, aid in access to testing, and promote self-quarantine as necessary [1]. While these efforts generally involve traditional direct outreach by public health workers, technologists and public health advocates have highlighted the potential of smartphone-based digital technologies to assist in contact tracing [2,3]. A May 2020 guidance from the Centers for Disease Control and Prevention (CDC) acknowledged that digital contact tracing tools for COVID-19 can function as a “supplement to traditional public health contact tracing with the use of voluntary opt-in proximity or exposure notification tools” [4]. 

The proliferation of digital contact tracing apps in response to COVID-19 builds on advancements in health informatics and the rise of health apps and eHealth over the past decade [5,6]. This expansion of digital health surveillance tools has a variety of potential benefits, including aiding in managing health conditions [7-9], modifying health behaviors [5,10], and helping facilitate racial equity in health information and health care [11,12]. Additionally, recent studies have found that digital health tools with exposure notifications are effective in decreasing infections from the coronavirus [13,14].

Despite the promise of digital surveillance tools to mitigate the spread of COVID-19, app-based contact tracing has had limited success in the United States [15,16]. As with all contact tracing approaches, the success of app-based contact tracing depends on individuals’ willingness to participate. Such willingness appears to be low [17,18]. In late 2020, Pew Research Center found that about 40% of Americans said they were unlikely to talk to contact tracers, and 50% said they were uncomfortable sharing location data from their phones [19].

Studies on public engagement with digital contact tracing around the world have proliferated since the start of the COVID-19 pandemic [13,20-24]. However, fewer studies have focused on the United States and drawn on representative survey samples. A notable exception is the work of Camacho-Rivera et al [18], who conducted a representative survey of US households between April and June 2020 to examine attitudes toward mobile health tools for COVID, including location-based exposure tracking. They found that although more than half of the respondents reported they were not likely to use mobile health tools for COVID, some sociodemographic and health-related factors were associated with more willingness to engage with digital contact tracing. For example, non-White respondents were more likely than their counterparts to say they were willing to use app-based location exposure tracking for COVID [18]. Additionally, systematic reviews of engagement with contact tracing during COVID (and some other contagious health outbreaks) across various countries have observed a number of barriers to uptake, including concerns about privacy and surveillance, mistrust of the government, and mistrust of technology [20,21]. Thus, while past research has pointed to several factors that influence contact tracing, limited research has considered how these factors interact in meaningful ways.

Gaining insights into differences regarding who is willing to participate in contact tracing may be especially consequential given the disproportionate impact of COVID-19 on racial/ethnic minority populations in the United States [25]. Greater willingness or hesitancy to participate in contact tracing among minoritized groups could affect the ability to stop the virus’ localized spread and could heighten the risks of morbidity and mortality in communities of color. Related research on the limited uptake of digital health technologies suggests that differences in uptake, especially along racial/ethnic and socioeconomic lines, can exacerbate health disparities [26,27]. For example, some studies on the adoption of telehealth technologies indicate that Black and Latino patients are less likely to use telehealth than White patients, limiting their access to care [28,29]. However, other studies have reported opposite findings, with Black and Latino patients being more likely to use telehealth than White patients [30,31]. Additionally, there is evidence that individuals with lower levels of education are less likely to use digital health platforms and services compared to those with more than a high school education [18,26,32].

As noted in other studies, willingness to use digital health technologies in general and app-based contact tracing in particular is likely undermined by issues of trust. Studies have demonstrated that individuals’ trust in the medical system influences their use of health services [33,34]. Lack of trust among racial/ethnic minorities, including lower trust toward medical providers, research, and treatments, is commonly cited as a key mechanism underlying disparities in health care [24,35-37]. Such differences in trust in the health care system may be further exacerbated by concerns around privacy and security, which can adversely affect patients’ willingness to use eHealth tools and provide accurate health information to providers [38,39]. Prior research shows that there are socioeconomic differences in trust, as adults with a college degree are more likely than those with lower educational attainment to report avoiding products or services because of privacy concerns [40]. Further, widespread uptake of contact tracing may be hampered by concerns regarding data privacy and trust in the government [20-22,41]. Recent evidence suggests that contact tracing efforts face greater resistance in communities where people have low levels of trust in public officials or are worried about state surveillance [42,43].

Given previous findings regarding the role of trust and mixed findings on the differential uptake of health technologies, including COVID-19 apps, we set out to determine the individual factors associated with the willingness to participate in digital contact tracing in response to COVID-19 in the US city of Detroit, Michigan. By focusing on Detroit, we highlight the attitudes of residents in a majority-minority city that experienced multiple waves of COVID-19 outbreaks and deaths since the start of the pandemic. In Detroit, Black residents have been disproportionately impacted by COVID-19, representing over 75% of known diagnoses and 90% of deaths, and they are 10 times more likely than White residents to have friends or family who have died from COVID-19 [44]. Using data from a population-based survey of Detroit, Michigan residents, we examined not only how the factors of race/ethnicity, education, and trust are associated with individuals’ willingness to download a contact tracing app during summer 2020, but also how those factors are intertwined in important ways. The findings of this study can help shed light on what factors need to be considered to encourage use of digital public health tools in the future, including how various subpopulations may respond to messages about or sources of those tools.

## Methods

### Data Source

To examine individual willingness to participate in app-based contact tracing, we analyzed data from 2 surveys conducted by the Detroit Metro Area Communities Study (DMACS) in 2020 (one in June and the other in July). DMACS is a panel study designed to regularly capture the perspectives and behaviors of a representative sample of Detroit adults [45,46]. DMACS launched in 2016 and recruited respondents from an address-based probability sample of Detroit households. It has refreshed its sample approximately once a year through additional address-based probability sampling. In response to the March 2020 declaration of COVID-19 as a national emergency and the emergence of Detroit as a “hotspot” of coronavirus cases, DMACS initiated a series of rapid response surveys on residents’ experiences with COVID-19 [44,47,48]. Due to restrictions on human subject interactions during the pandemic, participation in these surveys was limited to survey panelists who had provided email addresses or phone numbers prior to the COVID-19 pandemic. In the June survey (fielded between May 28 and June 11, 2020), 1802 panelists were invited and 1173 completed surveys (66.1% response rate). In the July survey (fielded between July 15 and July 29, 2020), 1772 panelists were invited and 1137 completed surveys (64.2% response rate). In both waves, surveys were either self-administered online by respondents or interview administered by telephone. We limited the current analysis to panelists who provided complete responses to the items measuring key independent and dependent variables in the June and July surveys. In total, our results reflected 1873 responses from 991 Detroit residents.

### Ethics Approval

Human subjects research was approved by the University of Michigan Health Sciences and Behavioral Sciences Institutional Review Board (HUM00187155).

### Measures

Our dependent variable captured respondent willingness to participate in app-based contact tracing with the item, “Would you be willing to download an app on your cell phone that would provide information to public health officials about close contact you have with other people?” Respondents could answer yes, no, don’t know, or “I do not own a cell phone.” Because our primary interest was to examine what distinguishes those who are willing to participate in app-based contact tracing, we dichotomized responses to compare those who said yes to those who said no or don’t know. We dropped the small number (n=18) of respondents who reported not owning a cell phone.

We examined the willingness to engage in app-based contact tracing in relation to self-reported race/ethnicity, gender, age, education, and income. We used multiple imputation with chained equations (MICE, implemented with Stata 17 software, StataCorp) and Rubin’s combination rules to fill in missing values of these and other independent variables to avoid dropping respondents from the analyses [49,50]. To ensure sufficient data coverage, we restricted our analyses to Detroit’s 3 largest racial/ethnic groups: non-Latino Black (hereafter Black), non-Latino White (hereafter White), and Latino. We defined “Latino” as any respondent who self-identified as being of “Hispanic, Latino, or Spanish origin,” including those who selected another racial identity and Latino. “White” and “Black” refer to respondents who selected only those respective categories (and no other ethnoracial categories) and who did not identify as Latino. Respondents who identified on the survey using other ethnoracial categories alone or in combination (Asian, Native American, Hawaiian or Pacific Islander, multi-race, or other) were omitted from this analysis owing to their small sample sizes and to avoid combining segments of unlike populations. Age was captured as a continuous variable. Categories were created for annual household income (<US $10,000, US $10,000-29,999, US $30,000-49,999, US $50,000-100,000, and >US $100,000). Because income was commonly missing and unwillingness to provide income information may be associated with data privacy concerns and unwillingness to provide other personal information relevant to contact tracing, we included a dummy variable in our analysis that captured whether the respondents withheld their income data. Education level (less than college degree, or college degree or higher) and gender (male or female) were captured as binary variables.

Finally, we included in our analyses several measures of respondents’ beliefs about and experiences with COVID-19. Specifically, we included a measure of respondent trust in the government (adapted from the ICF COVID Monitor Survey of US Adults [51]). The question asked was as follows: “How much do you trust the Michigan state government to deal with the COVID-19 pandemic, on a scale of 1 to 10, where 1 is not at all and 10 is entirely?” Responses were captured on a 10-point Likert scale and were mean-centered for interpretability. We focused on this measure of trust in the state government because our outcome variable made reference to sharing information with “public health officials,” and most local COVID health efforts and digital contact tracing tools were coordinated at the state level at this time. We also included, as dichotomous variables, measures of whether the respondent had a close friend or family member who died from the coronavirus; had a close friend or family member who got ill from the coronavirus; had been diagnosed with COVID-19; and would say the COVID-19 pandemic is very serious for them personally. Because our outcome variable was focused on the use of a contact tracing app, we included a binary variable for the availability of reliable internet access as a proxy for technological use and access. We also controlled for the effect of time by including a binary variable for survey wave. We did not use sampling weights because they are incompatible with the multiple imputation package of Stata 17 when estimating multi-level models, but we controlled for survey sampling strata and individual characteristics used in the creation of sample weights.

### Statistical Analyses

Because we were analyzing panel data with multiple observations per person, we used random effects logit models, which introduce a random intercept to help correct for person-level unobserved heterogeneity. We estimated a series of models to gain insights into respondents’ willingness to engage in app-based contact tracing. Our first model examined differences in the willingness to contact trace across a variety of individual attributes, including race/ethnicity, degree of trust in the government, and education. Our second and third models added interactions among these key variables to examine if the effects of trust and level of education vary by race/ethnicity of the respondent. We tested a 3-way interaction between race/ethnicity, education, and trust in the government but found that the results were substantively similar to the results for the interaction between education and race/ethnicity, and thus, we omitted these results for parsimony.

## Results

### Descriptive Statistics

Summary statistics for respondents are presented in Table 1. Less than half (826/1873, 44.1%) of the respondents reported they would be willing to participate in app-based contact tracing. Black residents comprised the majority of our sample (1486/1873, 79.3%), while 14.2% (265/1873) of our sample was White and 6.5% (122/1873) was Latino, similar to the proportions in the city of Detroit. On average, DMACS respondents reported a relatively high degree of trust in the state government to deal with COVID-19 (7.25 out of 10 points). The majority of respondents did not attend college (1301/1873, 69.5%) and had an annual household income below US $50,000 (1219/1772, 68.8%). The average respondent was 49 years old. Like many surveys, our sample had a higher proportion of female respondents (1372/1873, 73.3%). Our respondents reflected the severe impact of the COVID-19 pandemic on those living in Detroit, with 71.4% (1337/1873) saying that the pandemic was very serious for them personally and many saying that they had a close friend or family member who got ill (1088/1870, 58.2%) or died (745/1871, 39.8%) from COVID-19. However, only 5.5% (102/1868) of respondents said that they had been diagnosed with COVID-19 as of July 2020. In general, respondents were technologically connected, with 86.6% (1622/1873) reporting that they had reliable access to the internet at home.

**Table 1:** Descriptive statistics of the analytical sample.

Variable		Value (N=1873)
Willing to participate in contact tracing, n (%)		826 (44.1)
**Race/ethnicity, n (%)**		
	White	265 (14.2)
	Black	1486 (79.3)
	Latino	122 (6.5)
Trust in the state government (score range 0-10), mean (SD)		7.25 (0.25)
**Education, n (%)**		
	Less than college	1301 (69.5)
	College	572 (30.5)
**Gender, n (%)**		
	Male	501 (26.8)
	Female	1372 (73.3)
**Age (years)^a^**		
	Mean (SD)	48.96 (15.44)
	Range	18.16-93.43
**Income (US$)^a^, n (%)**		
	<10,000	380 (21.4)
	10,000-29,999	441 (24.9)
	30,000-49,999	398 (22.5)
	50,000-100,000	405 (22.9)
	>100,000	148 (8.4)
	Income missing	101 (5.4)
Family member died from COVID^a^, n (%)		745 (39.8)
Family member got ill from COVID^a^, n (%)		1088 (58.2)
Pandemic was very serious personally, n (%)		1337 (71.4)
Diagnosed with COVID^a^, n (%)		102 (5.5)
Internet use, n (%)		1622 (86.6)
**Wave, n (%)**		
	June 2020 (wave 9)	987 (52.7)
	July 2020 (wave 10)	886 (47.3)

^a^Variable imputed with multiple imputations. Within the data set of 1873 observations, income was missing in 101 cases, age was missing in 18 cases, information on whether a family member died from COVID was missing in 2 cases, information on whether a family member got ill from COVID was missing in 3 cases, and information on diagnosis of COVID was missing in 5 cases.

### Analytic Results

Results from our models are presented in Table 2. Model 1 examined the relationships of individuals’ demographics, trust in the government, and COVID-19 experiences with their willingness to download a contact tracing app. The results showed that individual characteristics were strong predictors of the willingness to participate in app-based contact tracing. Compared with White respondents, Black (odds ratio [OR] 0.45, 95% CI 0.23-0.86) and Latino (OR 0.32, 95% CI 0.11-0.99) respondents had significantly lower odds of being willing to participate in contact tracing. Similarly, the odds of women saying they would participate in app-based contact tracing (OR 0.44, 95% CI 0.27-0.70) were about half of the odds of men. The willingness to participate in contact tracing was associated with trust, whereby higher levels of trust in the state government’s management of COVID-19 were associated with greater odds of downloading a contact tracing app (OR 1.17, 95% CI 1.07-1.27). Furthermore, respondents who reported their household income had roughly 4 times the odds of being willing to participate in contact tracing as those who elected to not report their income (OR 0.24, 95% CI 0.09-0.63), which is perhaps a measure of the wariness about sharing one’s personal information. While it might be expected that individual experience with COVID-19 (being sick, having friends or family who have gotten sick or died from the virus, or feeling the pandemic is personally very serious) would increase the willingness to participate in preventative measures, including contact tracing, we found that only those who lost a close friend or family member to COVID-19 showed significantly greater odds of participating in app-based contract tracing (OR 1.72, 95% CI 1.12-2.62). Age, level of income, access to the internet, and time were not significant predictors of an individual’s willingness to participate in contact tracing via an app.

**Table 2:** Predictors of the willingness to participate in app-based contact tracing among the respondents.

Variable			Model 1^a^, OR^b^ (95% CI)		Model 2^a^, OR (95% CI)		Model 3^a^, OR (95% CI)
**Race/ethnicity**							
	White	Reference		Reference		Reference	
	Black	0.45 (0.23-0.86)		0.47 (0.24-0.92)		1.70 (0.67-4.30)	
	Latino	0.32 (0.11-0.99)		0.36 (0.12-1.10)		0.73 (0.21-2.62)	
Trust in the state government (centered)			1.17 (1.07-1.27)		2.14 (1.55-2.93)		1.16 (1.07-1.26)
**Race × trust**							
	White × trust	N/A^c^		Reference		N/A	
	Black × trust	N/A		0.51 (0.37-0.71)		N/A	
	Latino × trust	N/A		0.56 (0.36-0.87)		N/A	
**Education**							
	Less than college	Reference		Reference		Reference	
	College	0.73 (0.43-1.23)		0.73 (0.43-1.22)		6.12 (1.86-20.15)	
**Race × education**							
	White × college	N/A		N/A		Reference	
	Black × college	N/A		N/A		0.08 (0.02-0.27)	
	Latino × college	N/A		N/A		0.92 (0.98-1.01)	
**Gender**							
	Male	Reference		Reference		Reference	
	Female	0.44 (0.27-0.70)		0.43 (0.27-0.69)		0.44 (0.27-0.70)	
Age^d^			0.99 (0.97-1.00)		0.99 (0.98-1.00)		0.99 (0.98-1.00)
**Income (US$)^d^**							
	<10,000	Reference		Reference		Reference	
	10,000-29,999	1.47 (0.80-2.71)		1.47 (0.80-2.68)		1.54 (0.84-2.80)	
	30,000-49,999	0.79 (0.41-1.50)		0.79 (0.42-1.50)		0.79 (0.42-1.50)	
	50,000-100,000	0.65 (0.32-1.31)		0.61 (0.31-1.22)		0.69 (0.35-1.38)	
	>100,000	1.00 (0.37-2.72)		0.99 (0.37-2.67)		0.80 (0.30-2.18)	
	Income missing	0.24 (0.09-0.63)		0.23 (0.09-0.59)		0.28 (0.11-0.72)	
Family/friend died from COVID^d^			1.72 (1.12-2.62)		1.73 (1.14-2.64)		1.79 (1.17-2.73)
Family/friend got ill from COVID^d^			0.79 (0.53-1.18)		0.79 (0.53-1.17)		0.76 (0.51-1.13)
Pandemic was very serious personally			1.30 (0.88-1.91)		1.33 (0.90-1.96)		1.36 (0.92-2.01)
Diagnosed with COVID^d^			1.04 (0.46-2.32)		1.07 (0.48-2.38)		1.08 (0.49-2.40)
Internet use			1.33 (0.73-2.44)		1.27 (0.70-2.32)		1.38 (0.76-2.50)
**Wave**							
	June 2020 (wave 9)	Reference		Reference		Reference	
	July 2020 (wave 10)	0.89 (0.68-1.14)		0.88 (0.68-1.14)		0.88 (0.68-1.14)	
Constant			2.93 (0.44-19.45)		2.95 (0.45-19.17)		0.71 (0.10-5.18)

^a^Results from multilevel logistic regression with random effects. We controlled for sampling strata in our models, but we have not presented the nonsignificant effect of these strata to keep the table short. Data reflect 1873 responses nested within 991 respondents across 2 survey waves.

^b^OR: odds ratio.

^c^N/A: not applicable.

^d^Variable imputed with multiple imputation. Sensitivity analyses without imputation reach substantively similar conclusions as those of the imputed models.

To understand what may be driving racial/ethnic differences in the willingness to participate in contact tracing, subsequent models estimated interactions between race/ethnicity and trust (Model 2), and race/ethnicity and education (Model 3). By allowing the effects of race to differ by the degree of trust and level of education, we can better understand whether resistance to contact tracing among racial/ethnic groups is uniform or varies within subgroups.

**Figure 1:**
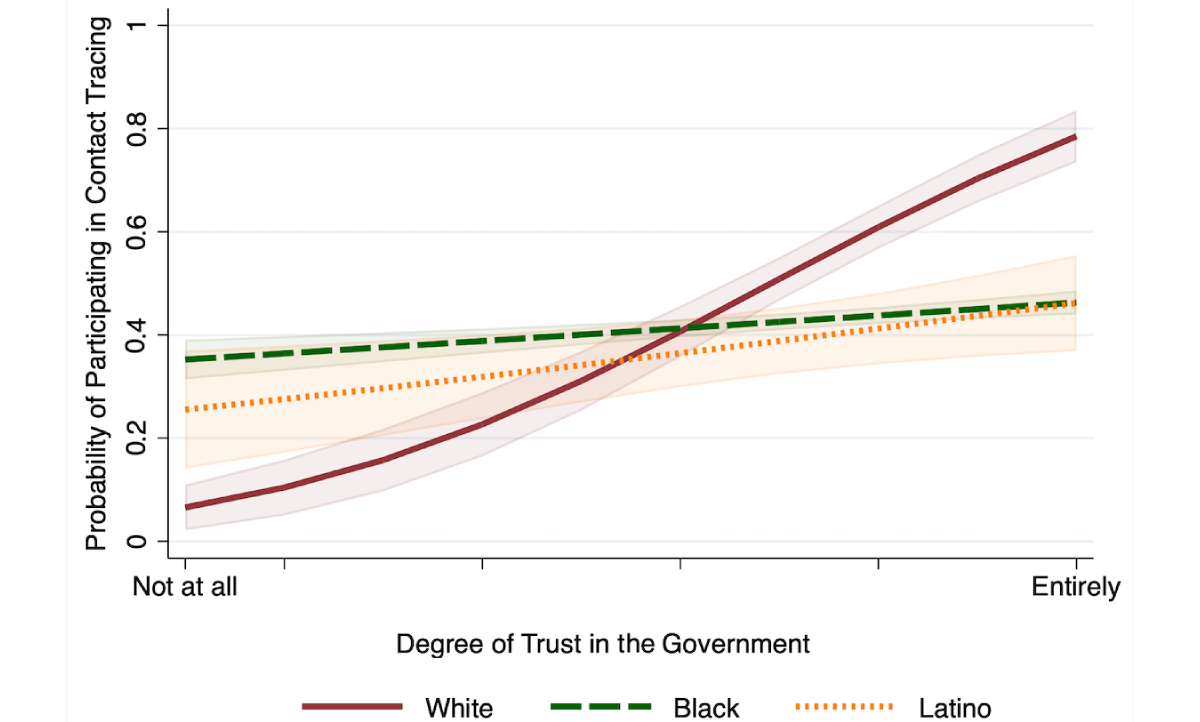
Predicted probability of the willingness to participate in app-based contact tracing by race and trust in the government.

Examining the interaction between race/ethnicity and trust (Model 2), we found that for White respondents, higher levels of trust in the state government’s management of COVID-19 were associated with greater likelihood of being willing to participate in contact tracing via an app (OR 2.14, 95% CI 1.55-2.93). This relationship was significantly weaker for Black and Latino respondents (Black: OR 0.51, 95% CI 0.37-0.71; Latino: OR 0.56, 95% CI 0.36-0.87). Figure 1 illustrates the relationship between race/ethnicity, trust, and contact tracing, showing the strong positive relationship between trust and the willingness to participate in contact tracing for White respondents and the negligible effect of trust on the willingness to participate in contact tracing for other respondents. Model 2 results also show that the effect of trust does not fully explain differences in the willingness to participate in contact tracing by race/ethnicity. Black respondents with an average degree of trust in the government remained significantly less likely than White respondents to be willing to engage in app-based contact tracing (OR 0.47, 95% CI 0.24-0.92). We also continued to find that women and those who did not report income were less likely to participate in contact tracing, while those who had a close acquaintance die of COVID remained more likely to participate.

**Figure 2:**
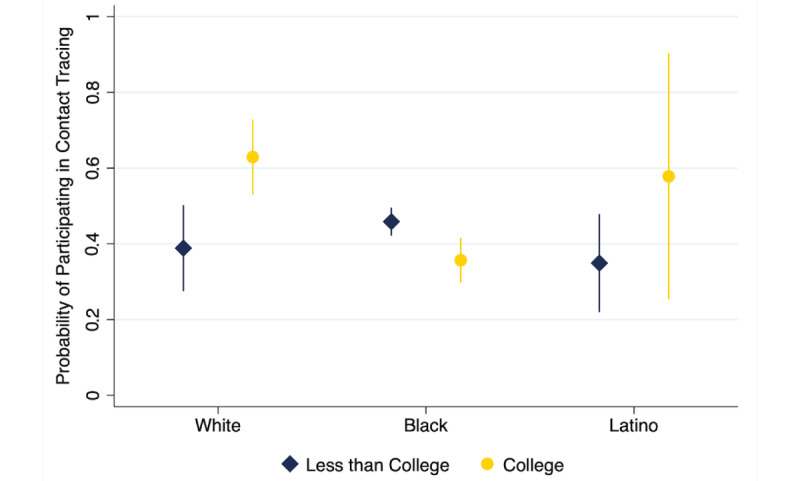
Predicted probability of the willingness to participate in app-based contact tracing by race and education level.

Turning to the interaction between race/ethnicity and education (Model 3), the main effects showed no significant difference in the willingness to participate in contact tracing among noncollege-educated respondents by race. However, the interaction showed that the effect of college education differs significantly depending on whether the respondent is Black or White. The diverging effect of education on the likelihood to engage in app-based contact tracing among White and Black respondents is illustrated in Figure 2. Among White respondents, the probability of participating in app-based contract tracing was significantly higher among college-educated respondents (predicted probability 0.63, 95% CI 0.53-0.73) compared to noncollege-educated respondents (predicted probability 0.39, 95% CI 0.28-0.50). However, among Black respondents, college-educated respondents were less likely to participate in app-based contact tracing (predicted probability 0.36, 95% CI 0.30-0.41) compared to noncollege-educated respondents (predicted probability 0.46, 95% CI 0.42-0.50). There was no significant difference between college- and noncollege-educated Latino respondents in the probability of participating in app-based contact tracing.

## Discussion

### Principal Findings

In this study, we found several factors that were associated with respondents’ willingness to participate in contact tracing using a digital app. Black and Latino respondents were significantly less likely than White respondents to say they are willing to use an app for contact tracing, while those with higher trust in the government as well as those who had a close family member or friend die of COVID-19 were significantly more willing to use a contact tracing app than those with low trust or no close experiences. We also found that the relationship between trust in the government and willingness to participate in contact tracing was much stronger for White respondents than for Black and Latino respondents among whom trust in the government does not matter as much for predicting the willingness to participate in contact tracing. Given the widespread evidence of mistrust in the medical system among racial/ethnic minorities [35-37], this lack of association between trust in the government and willingness to adopt app-based contact tracing among Black and Latino respondents may reflect a general apprehension around digital health care that cannot be moderated by the level of trust. At the same time, the effect of trust in the state government on White residents’ willingness or unwillingness to participate in contact tracing may reflect the partisan divide apparent nationally in response to COVID-19 [19].

In addition to our findings on trust, we also found that willingness to engage in app-based contact tracing varies substantially among racial/ethnic subpopulations depending on the level of education. While the likelihood of contact tracing among noncollege-educated respondents was relatively uniform across racial/ethnic groups, college-educated White respondents were substantially more likely to participate in contact tracing than noncollege-educated White respondents, and college- and noncollege-educated Black respondents. Moreover, for Black respondents, a college education significantly decreased the willingness to participate in contact tracing. In light of existing research indicating that education may influence one’s use of digital health platforms [26,32] and online products or services because of privacy concerns [40], the interaction between race and education regarding the willingness to participate in app-based contact tracing suggests an understudied phenomenon whereby education may amplify the fears of minority populations when it comes to public health and may mitigate the fears of White populations.

### Comparison to Prior Work

Our findings are consistent with prior research on public health and contact tracing apps, which has shown that factors, including personal experience with COVID-19, trust in the government, education level, and race and ethnicity, affect the willingness to use digital health tools [18,20,21]. Unlike previous work, however, we showed that some of these factors intersect in important ways. For example, while greater trust in the government was associated with increased willingness to use these digital tools among White respondents in our study, it had no significant effect among Black and Latino respondents.

Lower trust among racial and ethnic minorities is often linked to experiences of discrimination that reveal power imbalances and systemic inequalities in the practices of social institutions [52,53]. For example, systemic racism in health care is associated with disparities in access, quality of care, and health outcomes, as well as mistrust [54-59]. It is often racial minority individuals with higher levels of socioeconomic status who report more instances of discrimination in health care and in other institutions [52,55,60]. Upwardly mobile young adult Black and Latino individuals, for example, not only experience higher levels of discrimination than White individuals, but also are significantly more likely to experience acute and chronic discrimination compared to their socioeconomically stable counterparts [61]. Such research is consistent with our findings of an interaction between race/ethnicity and education, with college-educated Black respondents being significantly less willing to use a contact tracing app than either similarly educated White respondents or Black respondents with less than a college education. Awareness of and concerns about the extent of racial bias in digital technologies generally [62-66] may further contribute to reluctance to use these particular technologies among highly educated minoritized individuals.

Our findings are important because they suggest that factors that matter for some individuals and groups do not operate in the same way for others. Although trust in the government matters for the use of public health technologies for some individuals, it is likely to be interconnected with other social factors and conditions that must be considered. For most White individuals, trust in the government matters greatly, so understanding what influences that trust may be important for influencing the use of public health tools. For minoritized individuals, unequal experiences across social institutions may influence not only levels of trust but also more general opinions about and use of digital technologies. Future work must be attentive to not only how opinions, behaviors, and outcomes differ across minoritized groups, but also how actions and outcomes are associated with the lived experiences of individuals, stemming from institutionalized racism and other systems of inequality. 

### Limitations

A number of limitations should be considered when interpreting our findings. First, because the data were drawn from a single city, the findings may not be generalizable to digital contact tracing efforts in other communities or in the United States more broadly. Additional research is needed to understand the extent to which our findings on the willingness to participate in contact tracing extend to other communities with different demographic and socioeconomic compositions, including larger populations of other racial and gender minorities. As Detroit is a majority Black city in a majority White state, the effect of trust in the state government on individuals’ decisions to participate in contact tracing may be more pronounced here than in other communities. At the same time, Detroit’s experience, particularly early in the pandemic, as a COVID-19 hotspot may mean that residents are more willing to engage in contact tracing than other populations. Future research should investigate whether the patterns captured in our analysis hold in other communities and in the national population. Second, because our measure of contact tracing focused specifically on the willingness to download an app, it is possible that respondents’ hesitancy is shaped more by discomfort with or privacy concerns regarding phone apps or access to smartphones and not contact tracing specifically. Though we controlled for internet access as a proxy for technology access and use, and only examined responses among cell phone owners, future research would benefit from examining the willingness to participate in digital contact tracing across a variety of modalities. Finally, our data were collected within the first 4 months of the coronavirus pandemic being declared a national emergency in the United States. In the ensuing months and years, COVID-19 has continued to spread and people’s personal experiences of being infected with or knowing others infected with the coronavirus have changed. While digital contact tracing efforts in the United States have generally waned over time, it is possible that perspectives on contact tracing have shifted since the data were collected in response to the evolving nature of the pandemic and perspectives on public health generally.

### Conclusions

The impact of COVID-19 has been disproportionately experienced by people of color [25]. In Detroit, Black residents experienced extremely high levels of disease, death, and loss of loved ones as a result of the pandemic [44]. Contact tracing is an important public health tool to limit the spread of infectious diseases like COVID-19, and digital contact tracing apps can positively contribute to such efforts [3]. Yet, throughout the COVID-19 pandemic, people’s willingness to engage with digital contact tracing has varied [19].

Contact tracing technologies have the potential to advance public health efforts during the COVID-19 pandemic and beyond, but unequal uptake of these platforms across racial/ethnic groups and socioeconomic status may exacerbate the disparate impacts of health crises. Our findings show that residents’ wariness of participating in contact tracing is associated with trust in the government and education levels, suggesting that distrust and concerns about systemic inequality and surveillance may drive reluctance to use such tools, especially among people of color [24]. These results point to the importance of being attuned to the ways intersectional identities influence public health behaviors and the need to develop culturally informed advocacy when promoting these platforms in the future [23,67].

## Abbreviations

DMACS: Detroit Metro Area Communities Study
OR: odds ratio
